# Implementation of free breathing respiratory amplitude‐gated treatments

**DOI:** 10.1002/acm2.13253

**Published:** 2021-05-13

**Authors:** Susannah V. Hickling, Andrew J. Veres, Douglas J. Moseley, Michael P. Grams

**Affiliations:** ^1^ Department of Radiation Oncology Mayo Clinic Rochester MN USA

## Abstract

**Purpose:**

The purpose of this study was to provide guidance in developing and implementing a process for the accurate delivery of free breathing respiratory amplitude‐gated treatments.

**Methods:**

A phase‐based 4DCT scan is acquired at time of simulation and motion is evaluated to determine the exhale phases that minimize respiratory motion to an acceptable level. A phase subset average CT is then generated for treatment planning and a tracking structure is contoured to indicate the location of the target or a suitable surrogate over the planning phases. Prior to treatment delivery, a 4DCBCT is acquired and a phase subset average is created to coincide with the planning phases for an initial match to the planning CT. Fluoroscopic imaging is then used to set amplitude gate thresholds corresponding to when the target or surrogate is in the tracking structure. The final imaging prior to treatment is an amplitude‐gated CBCT to verify both the amplitude gate thresholds and patient positioning. An amplitude‐gated treatment is then delivered. This technique was commissioned using an in‐house lung motion phantom and film measurements of a simple two‐field 3D plan.

**Results:**

The accuracy of 4DCBCT motion and target position measurements were validated relative to 4DCT imaging. End to end testing showed strong agreement between planned and film measured dose distributions. Robustness to interuser variability and changes in respiratory motion were demonstrated through film measurements.

**Conclusions:**

The developed workflow utilizes 4DCBCT, respiratory‐correlated fluoroscopy, and gated CBCT imaging in an efficient and sequential process to ensure the accurate delivery of free breathing respiratory‐gated treatments.

## 1 INTRODUCTION

Respiratory motion management is an important strategy to account for breathing‐induced target motion during radiation therapy.^1^ If not considered during both the treatment planning and delivery processes, respiratory motion can lead to artifacts and target volumes that do not adequately encompass the target. Active respiratory motion management techniques can be used to minimize target motion during treatment delivery, allowing for a reduction in target volumes and improved normal tissue sparing. Such techniques include abdominal compression,^2^ breath hold,^3^ and free breathing phase or amplitude gating.^4^

Many thoracic and abdominal sites are susceptible to substantial motion, with abdominal and lower lung lesion motion often measured to be greater than 1 cm.^5^, ^6^ Inter‐ and intrafraction changes in target motion magnitude greater than 5 mm have been reported; thus, target motion analysis at time of simulation may not be adequate to account for changes in motion throughout the course of treatment.^7^, ^8^ Furthermore, changes in the relationship between external surrogate motion and target motion can occur throughout the course of treatment.^9^ Therefore, there is a need for patient‐specific motion analysis and a treatment delivery strategy that is robust to changes in a patient’s breathing pattern and target motion.

Respiratory‐correlated imaging is important to adequately account for target motion throughout the entire radiation therapy process.^1^ 4D computed tomography (4DCT) scans are often acquired at time of simulation when treating thoracic and abdominal sites to allow for accurate delineation of anatomy over the entire breathing cycle. Respiratory‐correlated fluoroscopy can be utilized to verify internal target volumes (ITV) for both gated and non‐gated treatment deliveries. The use of fluoroscopy for this purpose depends on target visibility and is most appropriate for large lung lesions or cases where there is a high‐density surrogate, such as fiducials. 4D cone beam computed tomography (4DCBCT) correlates CBCT projections with the measured respiratory trace to reconstruct volumetric images at different parts of the breathing cycle.^10^, ^11^ This reduces respiratory motion artifacts and offers improved verification of the ITV compared to 3D CBCT, which has been shown to underestimate the ITV in certain cases.^12^ Gated CBCT is another valuable pretreatment imaging technique that acquires CBCT projections in a predefined gating window. This is useful to mitigate motion artifacts and verify the gating thresholds prior to the delivery of gated treatments.^13^

Free breathing gating is a respiratory motion management strategy that allows patients to breathe normally during treatment while radiation is delivered during a predefined portion of the respiratory cycle. Phase‐ and amplitude‐based approaches have both been used to gate the beam to minimize target motion while radiation is delivered.^14^, ^15^ It has previously been suggested that such techniques require respiratory‐gated imaging to ensure accurate treatment delivery.^9^ As pretreatment imaging techniques advance there is the opportunity to improve the accuracy of free breathing gated treatment deliveries and account for changes in respiratory motion from time of simulation to treatment.

The implementation of free breathing gated treatment programs requires a dedicated commissioning process. However, there are few recommendations in the literature regarding workflow and commissioning best practices. TG‐142 offers several QA recommendations for gated systems including beam output and energy constancy, gate temporal accuracy, and surrogate calibration.^16^ Methodology to perform such tests has previously been reported in the literature.^17^ However, as gated treatment deliveries evolve with advances in pretreatment imaging, the tests performed during commissioning must follow suit.

The purpose of this work is to provide a practical guide to implementing free breathing amplitude‐gated treatments using 4DCT, 4DCBCT, gated CBCT, and respiratory‐correlated fluoroscopy. The workflow developed at our institution is described in detail, along with the steps taken to commission the technique. The pretreatment imaging process allows for the accurate delivery of free breathing amplitude‐gated treatments and is robust to changes in breathing and respiratory motion from time of simulation to treatment.

## 2 MATERIALS AND METHODS

### 2.A Workflow development

#### 2.A.1 Phantom

**Fig. 1:**
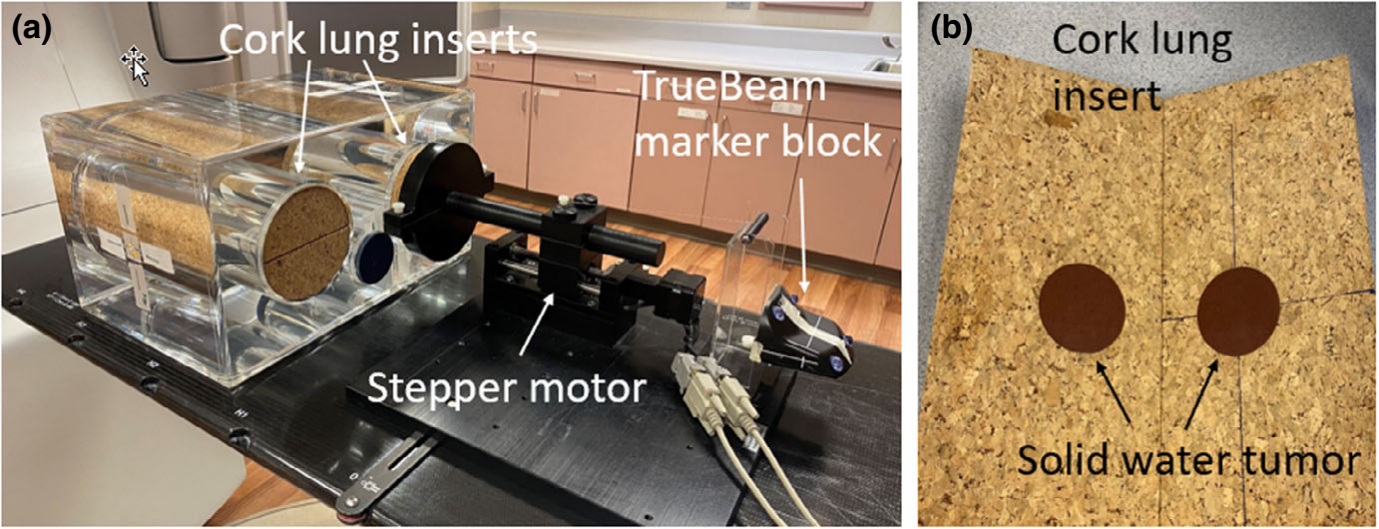
(a) Motion phantom consisting of two cork lung inserts inside of a water tank. A stepper motor attached to one of the lung inserts allows for programmable superior–inferior motion of the lung. (b) Lung insert containing a solid water tumor with an equivalent spherical diameter of 4.7 cm. Film is placed in a coronal plane through the center of the lung.

The workflow for the delivery of free breathing respiratory‐gated treatments was developed and commissioned using an in‐house lung motion phantom,^18^ shown in Fig. 1. The phantom consists of an acrylic water tank with two cork cylinders to represent lungs, one of which contains a solid water tumor with an equivalent spherical diameter of 4.7 cm. The cork lung is attached to a stepper motor (NSC‐A1, Newmark Systems, Rancho Santa Margarita, CA), and is capable of programmable superior–inferior motion using LabVIEW (National Instruments, Austin, TX). The respiratory marker blocks required to track breathing during CT acquisition and treatment delivery are attached to the motor, leading to a direct linear relationship between tumor and respiratory block motion. The cork lung is split to allow for film to be placed in a coronal plane, as shown in Fig. 1b.

#### 2.A.2 Motivation for amplitude gating

A key decision to make when developing a free breathing respiratory‐gated treatment program is whether phase‐ or amplitude‐based gating will be used. This decision is largely dependent on the capabilities of the available equipment and each technique’s ability to handle irregular respiratory traces. The Siemens Definition AS20 CT (Siemens Healthineers, Erlangen, Germany) scanners at our institution are equipped with the Varian RPM system (Varian Medical Systems, Palo Alto, CA) and generate phase‐based 4DCT scans. TrueBeam linear accelerators (Varian Medical Systems, Palo Alto, CA) have the capability of both phase and amplitude‐gated treatment delivery. The TrueBeam can use a predictive filter to monitor breathing regularity during treatment and halt treatment delivery if breathing periodicity exceeds a user configurable threshold. Predictive filter options range from 0% (predictive filter not used) to 100%, with the system default set to 20%.

**Fig. 2:**
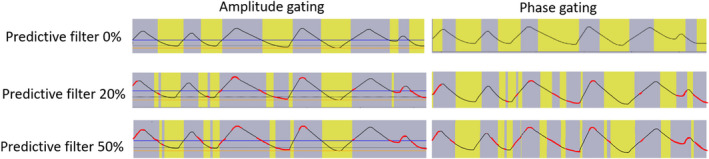
Evaluation of the predictive filter using both amplitude and phase gating (20%–70%) thresholds with an irregular trace. Red parts of the trace correspond to when the predictive filter recognizes irregular breathing periodicity. Yellow indicates the portion of the breathing trace when radiation is delivered.

Amplitude and phase‐gated treatment deliveries were assessed by programming an irregular breathing trace for the in‐house motion phantom. Phase gating thresholds of 20–70% and the corresponding amplitude gate thresholds were both evaluated. Fig. 2 displays screenshots with different predictive filter settings for both phase‐ and amplitude‐based gating. Red portions of the trace indicate irregularity as determined by the predictive filter, resulting in a pause in treatment delivery. Yellow regions correspond to when radiation is being delivered. It is observed that phase‐gated treatment deliveries result in the delivery of radiation near maximum inhale for the case of this irregular trace. Additionally, using the predictive filter results in a low duty cycle for both amplitude and phase‐gated treatments. This evaluation motivated the use of amplitude‐gated treatment deliveries with the predictive filter disabled to ensure that radiation is consistently delivered at patient exhale in the case of irregular breathing.

### 2.B Clinical workflow

The clinical workflow implemented at our institution utilizes a marker block placed on the patient’s abdomen as a surrogate of respiratory motion. A 4DCT is acquired at time of simulation and a phase‐based reconstruction allows for analysis of target motion throughout the breathing cycle. A subset of phases over exhale where target motion is minimized to an acceptable amount is chosen for planning and treatment. Pretreatment imaging defines and verifies amplitude gate thresholds to correspond to the planning phases and an amplitude‐gated treatment is delivered. This workflow has been clinically applied to a variety of disease sites at our institution, including lung, liver, cardiac, and abdominal cases. It is described below in detail and illustrated using a representative clinical lung case that was treated to 60 Gy in eight fractions.

#### 2.B.1 Simulation

A Siemens Definition AS20 CT scanner (Siemens Healthineers, Erlangen, Germany) is used to acquire a 4DCT scan at simulation. The respiratory trace is recorded using the Varian RPM system (Varian Medical Systems, Palo Alto, CA) to obtain a phase‐based reconstruction over ten respiratory phase bins. Target motion is evaluated using a workflow created in MIM (MIM Software Inc., Beachwood, OH) by contouring the target or other structure of interest on the 50% phase and propagating the contour to other phases. A plot showing the change in contour centroid position as a function of phase is generated for each dimension. This allows motion to be evaluated throughout the respiratory cycle to determine a subset of phases over exhale that minimize motion to an acceptable amount, while considering duty cycle. Note that this can also be done by simply stepping through the 4DCT phases and manually measuring target/surrogate displacement; thus, the use of MIM is not required.

**Fig. 3:**
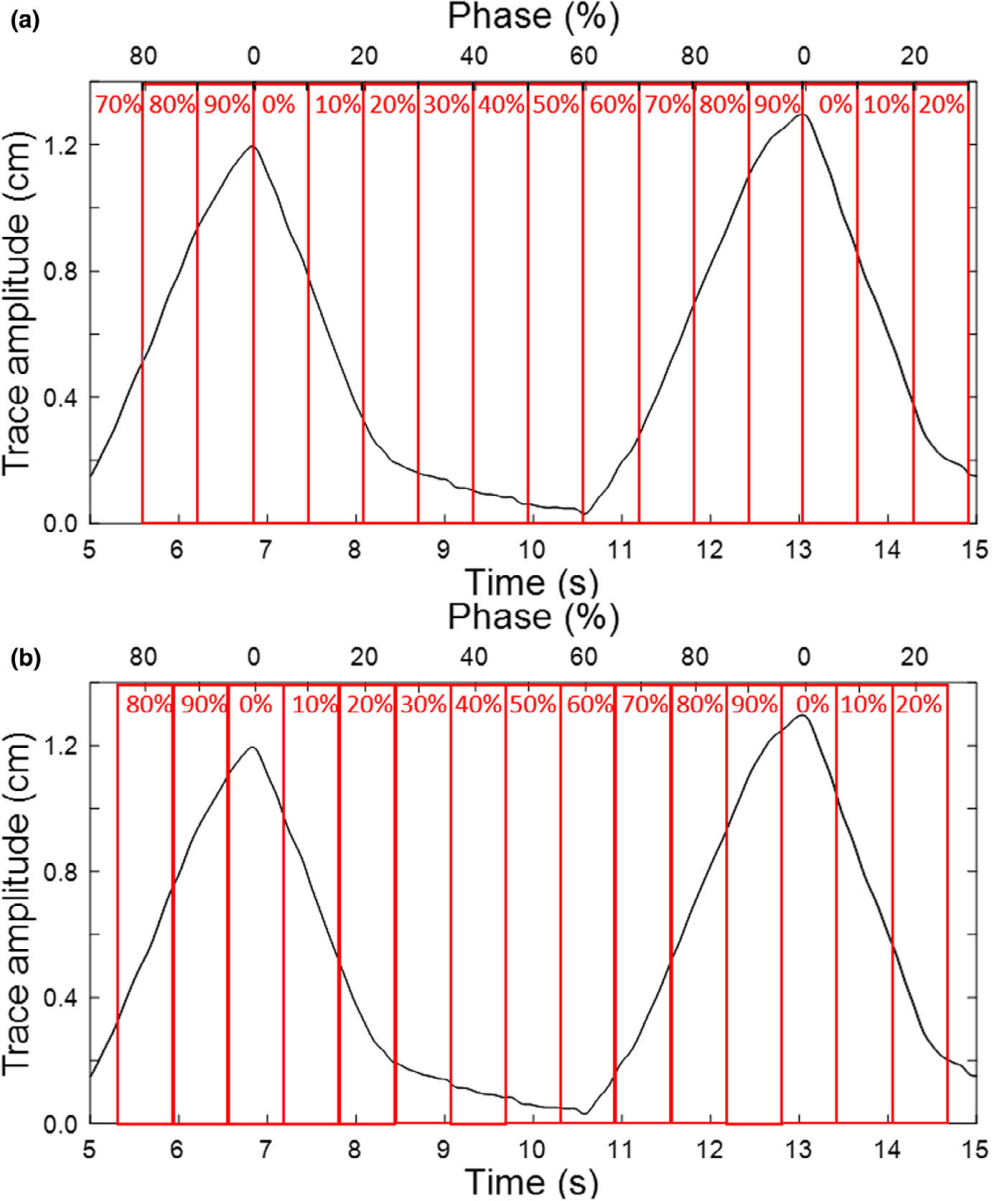
Example respiratory trace and the corresponding mapping of the temporal axis into phase (%). For each respiratory cycle, phase indicates the temporal position relative to maximum inhale, which is denoted as 0%. The different phase binning conventions for (a) 4DCT and (b) 4DCBCT are illustrated.

It is important to understand the conventions that the 4D modalities use to label phases. The CT scanner used in this work defines 0% to be maximum inhale and reconstructs phases in ten 10% bins that are labeled as the *minimum* phase in that bin. For example, the 0% phase bin dataset includes phases 0%–9%, the 10% phase bin dataset ranges from phases 10% to 19%, the 90% phase bin dataset contains phases 90%–99%, and so forth. This is illustrated in Fig. 3a.

**Fig. 4:**
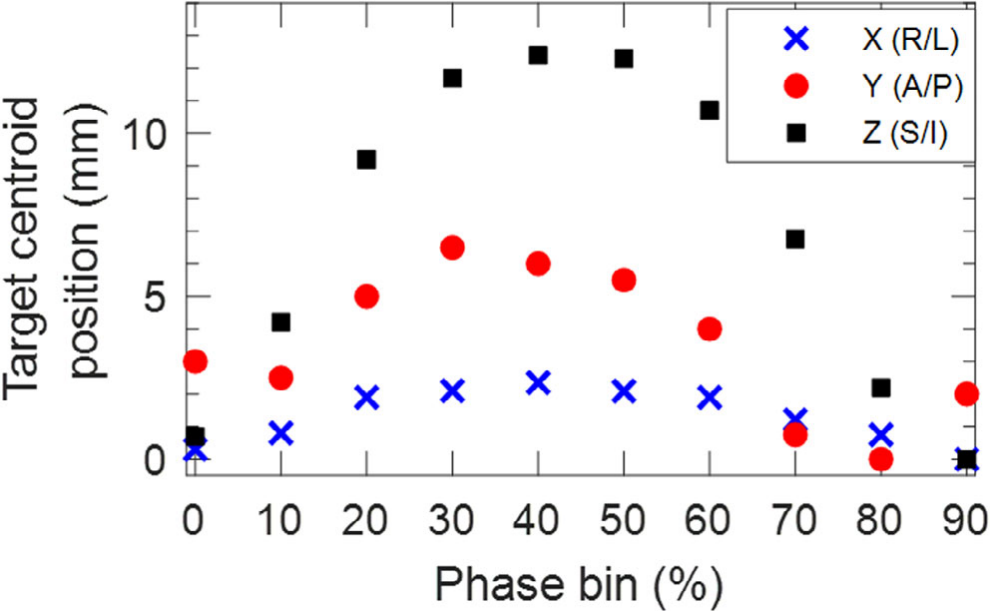
Target motion analysis from the planning 4DCT dataset for a clinical lung case. In this example, phase bins 20%–60% are chosen for planning to minimize right–left motion to 0.5 mm, anterior–posterior motion to 2.5 mm, and superior–inferior motion to 3.2 mm.

An example of 4DCT motion analysis is shown in Fig. 4 for the clinical lung case. There is a total of 2.5 mm, 6.5 mm, and 12.5 mm of motion in the right–left, anterior–posterior, and superior–inferior directions, respectively. Since superior–inferior motion dominates, we first look at the target centroid positions in that direction to determine which phases to treat over. Over the phase interval 20–60%, the minimum target centroid position is 9.2 mm (at phase 20%) and the maximum is 12.4 mm (at phase 40%). Thus, the residual superior–inferior motion over phases 20–60% is the difference between these two points, or 3.2 mm. In the right–left and anterior–posterior directions, the residual motion over phases 20%–60% is 0.5 mm and 2.5 mm, respectively. This amount of motion was deemed acceptable by the physician; thus, in this case, the 4DCT phase bins 20%–60%, which encompass the exhale portion of the respiratory cycle, were chosen for planning/treatment. This corresponds to individual phases 20%–69%.

#### 2.B.2 Treatment planning

**Fig. 5:**
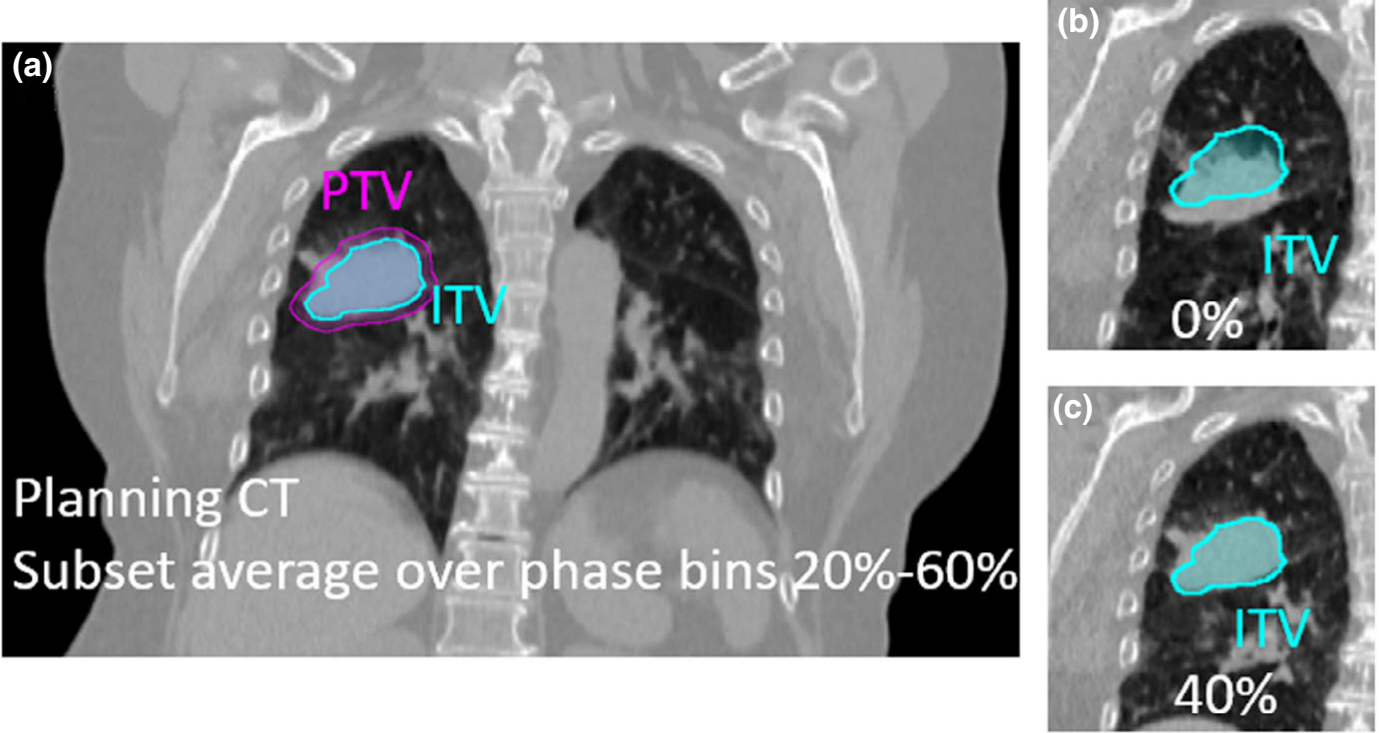
Clinical lung case example planned on phase bins 20–60%. (a) The ITV indicates the position of the target over the planning phases. (b) The target is observed to be outside the ITV contour at phase 0% (as expected since it corresponds to maximum inhale) and (c) inside the ITV contour at phase 40% (as expected since 40% falls within the planning phases).

A phase subset average CT over the exhale phases found to minimize motion is generated for treatment planning. For this clinical lung case example, the planning CT was generated over phase bins 20%–60%. During planning, a “tracking structure” is contoured to indicate the position of the target or a suitable surrogate over the planning phases. Ideally this structure should be visible on fluoroscopy to guide setting the amplitude gate thresholds during pretreatment imaging. For lung cases, this tracking structure is the target ITV over the planning phases. For abdominal cases where the target is not expected to be well defined on pretreatment imaging, fiducials, a stent, or the diaphragm can be used as a surrogate. For an object to be a useful surrogate, it must have the same temporal motion characteristics as the target and a fixed relative location. This is assessed by evaluating the surrogate and target motion on the 4DCT and through clinical experience. For cases where a surrogate is used the tracking structure is the surrogate ITV over the planning phases. Since the target in this example lung case is easily distinguishable, the tracking structure is the ITV over phase bins 20–60%, as shown in Fig. 5.

#### 2.B.3 Pretreatment imaging

A Varian TrueBeam v2.7 (Varian Medical Systems, Palo Alto, CA) linear accelerator is used for treatment and the TrueBeam reflector block is placed on the patient’s abdomen to record a respiratory trace. The system first learns the patient’s breathing trace over four respiratory cycles to set a baseline. Any couch movement greater than 2 mm causes the system to relearn and re‐baseline the breathing trace.

**Fig. 6:**
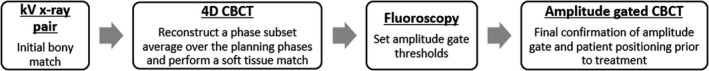
The pretreatment imaging workflow used for alignment and to set the amplitude gate thresholds for treatment.

The pretreatment imaging workflow is designed to determine and verify the amplitude gate corresponding to when the target or surrogate is inside the tracking structure generated during planning. Fig. 6 summarizes the main steps of the pretreatment imaging workflow.

The first imaging step is an orthogonal kV x‐ray pair to provide an initial bony alignment. Next, a 4DCBCT scan is acquired. Using default settings, the 4DCBCT takes 2 minutes to acquire and 90 seconds to reconstruct. The 4DCBCT scan acquires projections throughout the respiratory cycle and, by default, reconstructs ten 10% phase bins. Maximum inhale is defined as 0%, and each phase dataset is labeled as the *middle* phase in that bin. For example, the 0% phase bin dataset includes phases 95%‐4%, the 10% phase bin dataset ranges from phases 5% to 14%, etc. This is illustrated in Fig. 3b.

There are two 4D reconstruction algorithms available with the TrueBeam software: an “Advanced 4D” algorithm using a McKinnon‐Bates reconstruction^19^ and a “Basic 4D” algorithm. Briefly, the advanced algorithm uses all projections to reconstruct one 3D volume which is then forward projected to create difference projections that are compared to the acquired projections. The presence of motion causes dissimilarities between the difference and acquired projections, which are then added to the prior image for each phase bin dataset. The “Basic 4D” algorithm simply bins the acquired projection into each phase bin and reconstructs each phase bin image separately. It is common for 4DCBCT data to be undersampled in order to achieve a reasonable scan time, which comes at the cost of streaking and view aliasing artifacts. The “Advanced 4D” algorithm performs better than the “Basic 4D” algorithm on undersampled data.^19^ Unless otherwise specified, all 4DCBCT scans acquired in this work used the “Advanced 4D” algorithm.

After the acquisition of the 4DCBCT, a subset average CBCT is reconstructed over the planning phases to match to the planning CT. Due to the different method of phase binning between the 4DCT and 4DCBCT imaging systems, if the planning phase bins are x%–y%, the 4DCBCT average is reconstructed over phase bins (x + 10)%‐y%. In the clinical lung example, the planning CT is over phase bins 20%–60% (which is individual phases 20%–69%) and the average CBCT for matching is reconstructed over phase bins 30%–60% (which is individual phases 25%–65%). If the 20% phase bin was included in the 4DCBCT phase subset average this would include phases 15–19%, which are outside of the planning phases.

**Fig. 7:**
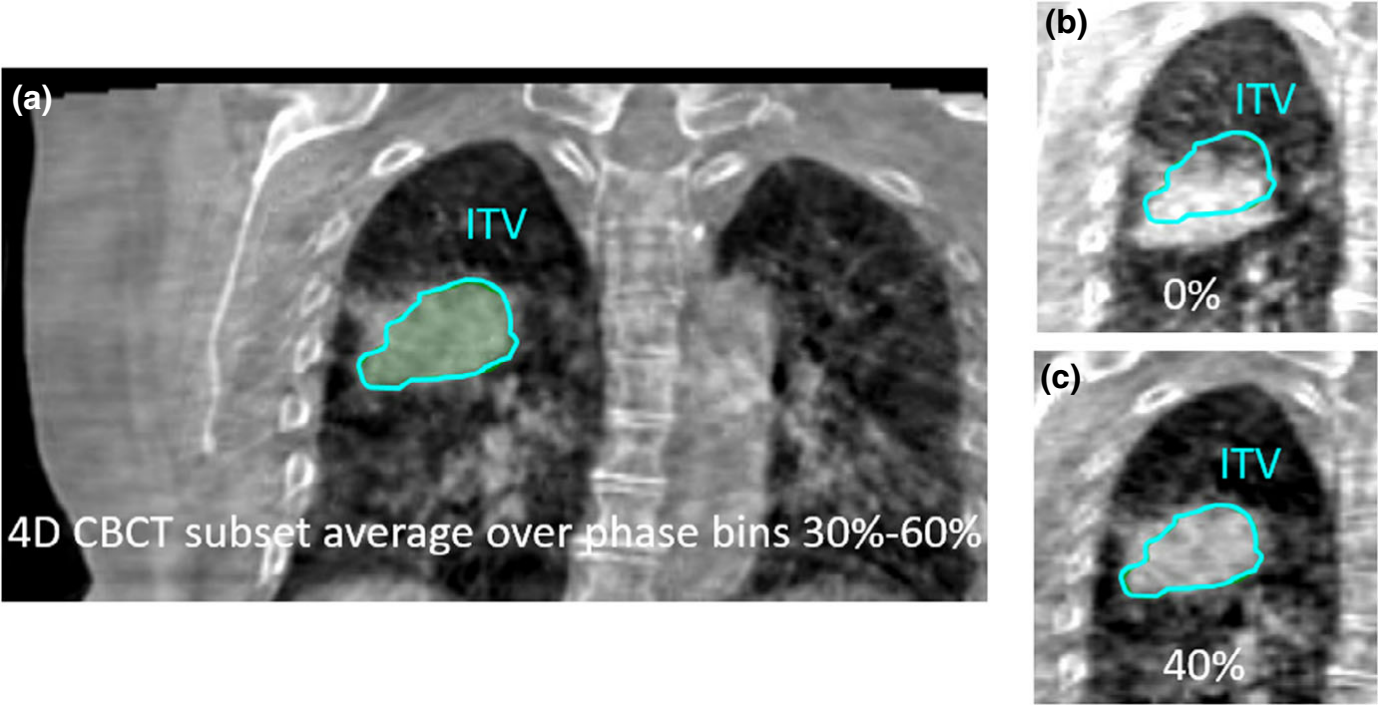
Coronal 4DCBCT images for the clinical lung case. (a) A 4DCBCT subset average over phase bins 30–60% is reconstructed for initial matching. (b) The 4DCBCT movie loop shows the target outside of the ITV in the 0% phase bin and (c) inside the ITV in the 40% phase bin, as expected.

A soft tissue match^20^ is performed between the planning CT and the 4DCBCT subset average over the planning phases. Fig. 7 displays 4DCBCT image slices for the clinical lung case. In Fig. 7a, it can be seen that the target is entirely in the ITV on the 4DCBCT subset average, as expected. After this match, the 4DCBCT movie loop workspace is used to step through and assess target motion over all ten phase bins. Reviewing the 4DCBCT movie loop is valuable to determine if target/surrogate motion is consistent with motion at the time of simulation by observing during which phases the target/surrogate is inside and outside the tracking structure. Figs. 7b and 7c show that the target is outside the tracking contour (ITV) at inhale (0% phase bin) and inside at exhale (40% phase bin), as expected.

**Fig. 8:**
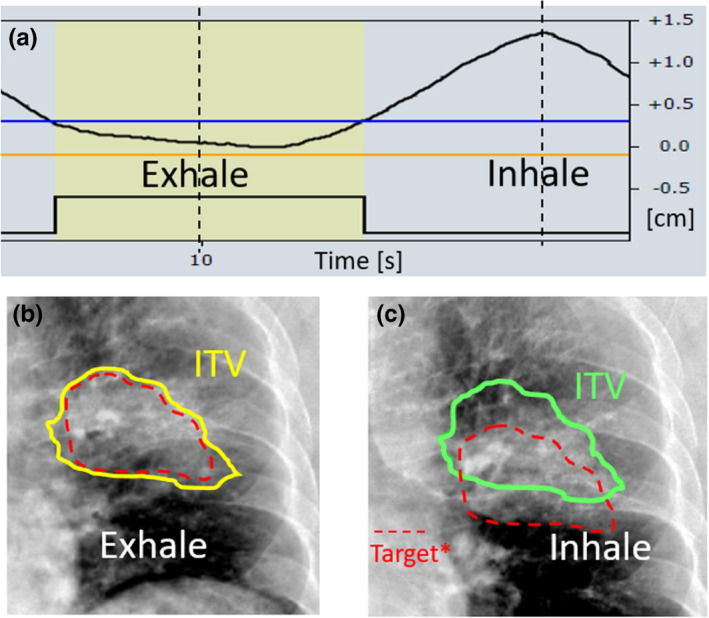
Posterior–anterior fluoroscopy images for the clinical lung case. (a) The acquisition of fluoroscopy images is correlated with the respiratory trace. (b) At exhale, the target is in the ITV tracking contour and the amplitude gate is set to include this part of the trace, causing the contour to turn yellow. (c) At inhale, the target is outside of the ITV tracking contour, as expected. The contour is green, indicating that the image was acquired during a part of the respiratory cycle outside of the amplitude gate. *Note that the dashed red line represents the target, and is included here for illustrative purposes only. This contour is not visible at the machine.

Next, kV fluoroscopy is used to determine the amplitude gate thresholds that will be used for treatment delivery. Anterior–posterior and/or lateral angles are used for fluoroscopy, depending on the primary direction of motion observed at time of simulation and 4DCBCT analysis. Fluoroscopy images are acquired for approximately three breathing cycles and reviewed to determine the amplitude gate thresholds. This review occurs after the acquisition of fluoroscopy images in a separate workspace where the position of each image frame is shown relative to the point of the respiratory trace at which it was acquired. This is visualized in Fig. 8 for the clinical lung case. Using the tracking structure, which in this case is the ITV, the amplitude gate thresholds are adjusted such that the target is inside the tracking structure during the gate (contour turns yellow as in Fig. 8b) and the target is outside of the tracking structure outside of the gate thresholds (contour is green as in Fig. 8c). Evaluating when the target is in the tracking structure is a manual process and is determined jointly by the radiation oncologist, physicist, and radiation therapist. The same principle applies if a surrogate (e.g. fiducials, diaphragm) is used for motion assessment instead of the target.

The final imaging prior to treatment is an amplitude‐gated CBCT. This utilizes the amplitude gate thresholds set from fluoroscopy to acquire CBCT projections when the patient’s respiratory trace is within the amplitude gate. This confirms both the amplitude gate and the soft tissue match from the 4DCBCT. If the target or surrogate blur is larger on the amplitude‐gated CBCT than on the planning CT, this implies that the amplitude gate may not be appropriate and the fluoroscopy images should be reviewed again or repeated. If shifts are required to match the gated CBCT to the planning CT, this is indicative of a poor 4DCBCT soft tissue match or patient movement, and it is recommended to shift and verify with a second gated CBCT.

#### 2.B.4 Treatment delivery

The patient’s breathing is monitored closely during treatment. Treatment is manually paused if there are large negative baseline drifts in the patient’s respiratory trace that result in the inhale being inside the amplitude gate. If there is a persistent baseline drift that does not recover, the breathing trace is relearned and a gated CBCT is taken to determine if the change in respiratory trace baseline correlates with a change in target position or motion. In the event, the resulting gated CBCT to planning CT match is poor, fluoroscopy to re‐adjust the amplitude gate thresholds or a patient shift are considered.

### 2.C Commissioning measurements

**Fig. 9:**
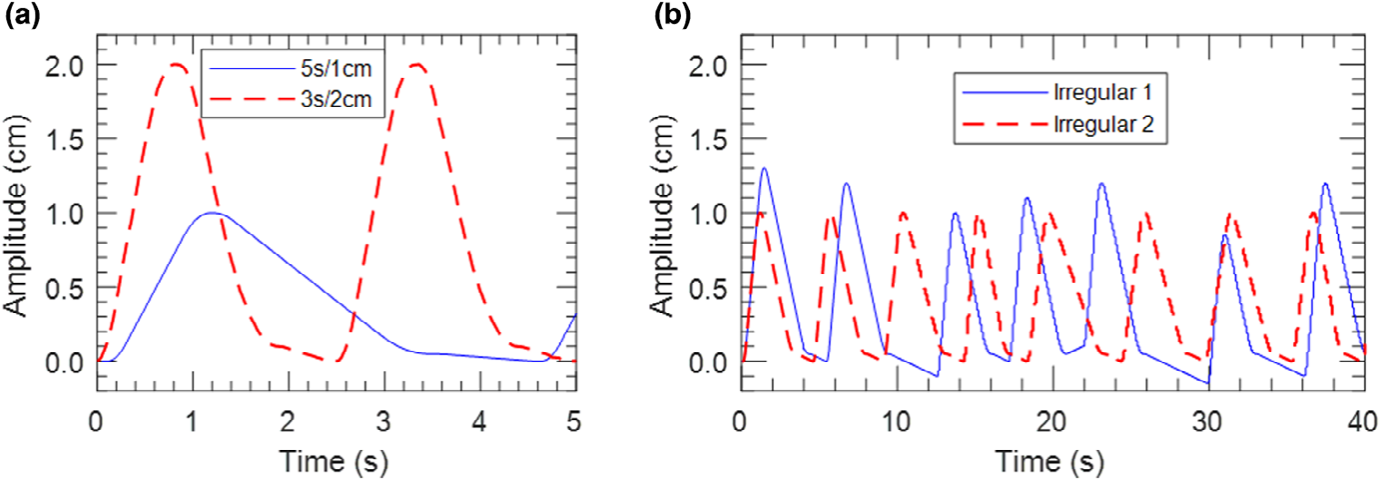
(a) Periodic 5 s/1 cm and 3 s/2 cm traces used for commissioning. (b) Irregular breathing traces based off of the nominal 5 s/1 cm trace.

The lung motion phantom described in Section 2.A. was used for commissioning this technique. Two periodic breathing traces, shown in Fig. 9a, were programmed for commissioning. One trace was considered to represent typical respiratory motion with a period of 5 s and amplitude of 1 cm (5 s/1 cm), while the other trace has a faster breathing period of 3 s and amplitude of 2 cm (3 s/2 cm). Two irregular traces based off the nominal 5 s/1 cm trace were also programmed, as seen in Fig. 9b.

#### 2.C.1 4DCBCT commissioning

Evaluating the functionality of 4DCBCT was a key part of our commissioning process. Since this workflow requires using multiple systems to acquire 4D scans, it is crucial to understand and verify how each is binning phases. As discussed in Sections 2.B.1 and 2.B.3 and illustrated in Fig. 3, the 4DCT and 4DCBCT systems used in this work both divide the respiratory cycle into ten phase bins, but bin phases in different ways. To verify proper binning of the phases, the 4DCT and 4DCBCT images acquired using the lung phantom with both the 5 s/1 cm and 3 s/2 cm traces were evaluated to determine the position of the target at each phase. The target position was defined as the center position between the superior and inferior boundary of the target.

An important component of the pretreatment imaging process is evaluation of motion from the 4DCBCT scan. Several measurements were made with the lung motion phantom and different programmed traces to understand the accuracy and limitations of motion assessment from 4DCBCT imaging. For each case, total motion was measured as the difference between the superior edge of the tumor at maximum inhale and exhale.

Varian indicates that the “Advanced 4D” algorithm does not correctly represent the motion of radio‐opaque fiducial markers, and therefore recommends using the “Basic 4D” algorithm for such cases. This was verified by placing two fiducials 2 cm apart in a cork lung insert and using the 5 s/1 cm trace to acquire 4DCBCT scans reconstructed with both available algorithms.

#### 2.C.2 End to end testing

For end to end testing, the entire workflow from simulation to treatment delivery was followed for both the 5 s/1 cm and 3 s/2 cm periodic breathing traces. Motion was evaluated and a phase subset average planning CT over phase bins 20–70% was reconstructed for each case, minimizing motion to 0.4 cm and 0.8 cm over the treated phases for the 5 s/1 cm and 3 s/2 cm cases, respectively. An ITV was contoured over the 20–70% phase subset average, with a 5 mm margin added to form the PTV. Simple AP/PA plans using static MLC‐shaped 6 MV beams were developed to deliver 2 Gy to the PTV. These 3D plans were used to mitigate potentially confounding interplay effects present in VMAT plans. The pretreatment imaging workflow described in Section 2.B.3 was followed using the ITV as the tracking structure to determine amplitude gate thresholds.

Film measurements were performed with Gafchromic EBT3 film and analyzed with FilmQA Pro software (Ashland, Bridgewater, NJ, USA) following a previously published single scan, triple channel dosimetry protocol.^21^ Film was placed inside of the cork lung insert in a coronal plane through the center of the solid water lesion. Gamma analysis was used to compare film measurements to the planned dose distributions.^22^

## 3 RESULTS

### 3.A 4DCBCT commissioning

**Fig. 10:**
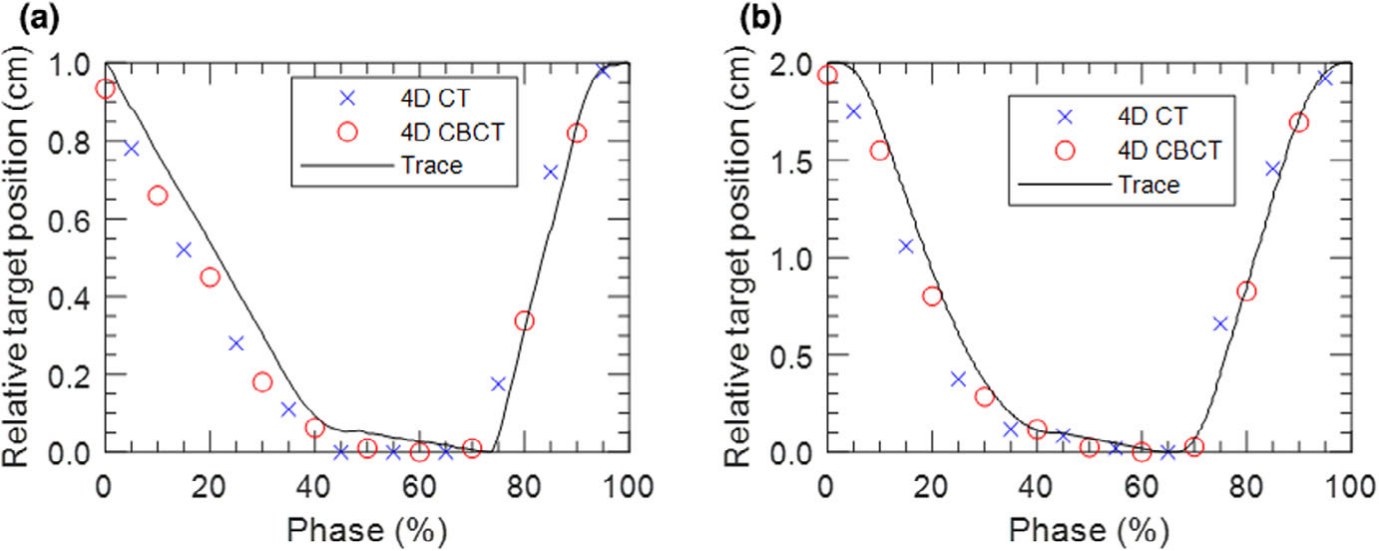
The central position of the target plotted at the midpoint of each phase bin from 4DCT and 4DCBCT images for the (a) 5 s/1 cm and (b) 3 s/2 cm traces.

Fig. 10 displays the position of the target in the lung phantom as a function of the midpoint of each 4DCT/4DCBCT phase bin along with the programmed breathing trace. This shows that 4DCT and 4DCBCT imaging correctly display target position relative to each other, accounting for the appropriate 5% offset between phase binning conventions. Note that perfect agreement with the trace is not expected due to uncertainties with determining the boundaries of the target, voxel size, and motor movement.

Table 1 displays the motion measured from the 4DCBCT scan for a variety of different traces. It is observed that measured motion agrees well with the programmed value for periodic traces with periods ranging between 3 s and 10 s. Note that perfect agreement with input motion is not expected since each phase bin image is the average over 10% of the respiratory cycle. Traces with exhale pauses of 20 s and 40 s half way through 4DCBCT acquisition were investigated since occasionally patients take an extended exhale or fall asleep during treatment. A pause of 20 s accounts for 17% of the 2‐minute 4DCBCT acquisition, and resulted in a measured motion within 0.5 mm of the corresponding periodic trace. Extending the exhale pause to 40 s resulted in the measurement of very little motion. Thus, it is recommended that 4DCBCT acquisition is suspended in the event of a pause in breathing that exceeds 20 s.

**Table 1:** Measured motion from 4DCBCT scans acquired with the lung phantom for different programmed breathing traces.

Trace description	Measured motion (cm)
Amplitude 1 cm, period 5 s	0.95
Amplitude 2 cm, period 3 s	1.94
Amplitude 1 cm, period 10 s	0.93
Amplitude 1 cm, period 5 s, pause for 20 s at exhale	0.90
Amplitude 1 cm, period 5 s, pause for 40 s at exhale	0.05

**Fig. 11:**
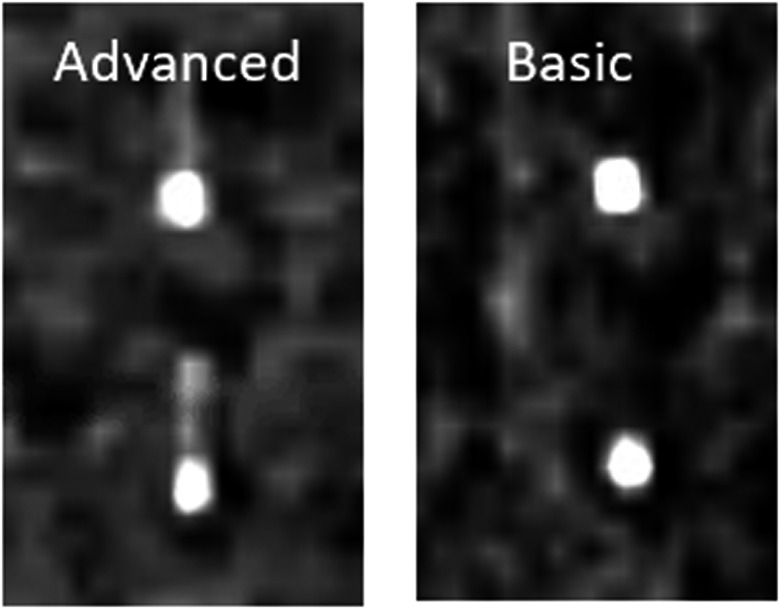
Coronal slices from the 4DCBCT 50% phase bin image of two fiducials placed 2 cm apart in a lung insert reconstructed using the advanced 4D and basic 4D algorithms.

Fig. 11 displays coronal slices from the 50% phase bin image of 4DCBCT scans reconstructed using the “Advanced 4D” and “Basic 4D” algorithms. The fiducial shape is not preserved on the “Advanced 4D” reconstruction, making it difficult to determine fiducial position and measure motion. Thus, the “Basic 4D” algorithm should be used for cases where fiducials are acting as a surrogate of target motion.

### 3.B End to end testing

#### 3.B.1 Film to plan comparison for two periodic traces

**Fig. 12:**
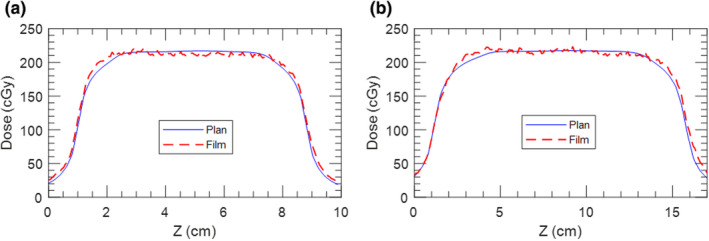
Plan and film superior–inferior profile comparison for the (a) 5 s/1 cm case and (b) 3 s/2 cm case.

Fig. 12 displays the end to end test results for the 5 s/1 cm and 3 s/2 cm traces. Superior–inferior profiles through the center of the target were extracted from film measurements and compared to the plan, as shown in Fig. 12a and Fig. 12b for the 5 s/1 cm and 3 s/2 cm cases, respectively. Film and planned dose distributions were also compared using gamma analysis with a pass rate criteria of 2%/2 mm and minimum threshold of 10%. The gamma pass rates were 98.6% and 98.5% for the 5 s/1 cm and 3 s/2 cm plans, respectively.

#### 3.B.2 Interuser variability

**Fig. 13:**
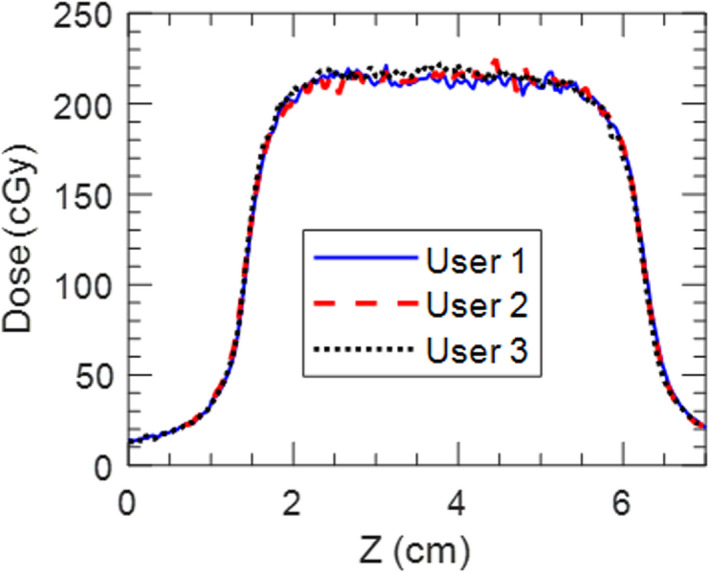
13Superior–inferior profiles through the center of the target from film measurements of the 5 s/1 cm plan done by three different users.

Interuser variability of the workflow was evaluated by having three independent users perform the pretreatment imaging and treatment delivery process with the 5 s/1 cm trace. While there is potential subjectivity in setting amplitude gate thresholds from the fluoroscopy images, the upper gate thresholds set by all three users were within 0.9 mm (4.1 mm, 4.5 mm, and 5.0 mm). Fig. 13 shows superior–inferior profiles extracted from each user’s film measurement. When 2D film measurements from each user were compared to each other all combinations agreed with 2%/2 mm gamma pass rates of 99%.

#### 3.B.3 Robustness to irregular breathing

**Fig. 14:**
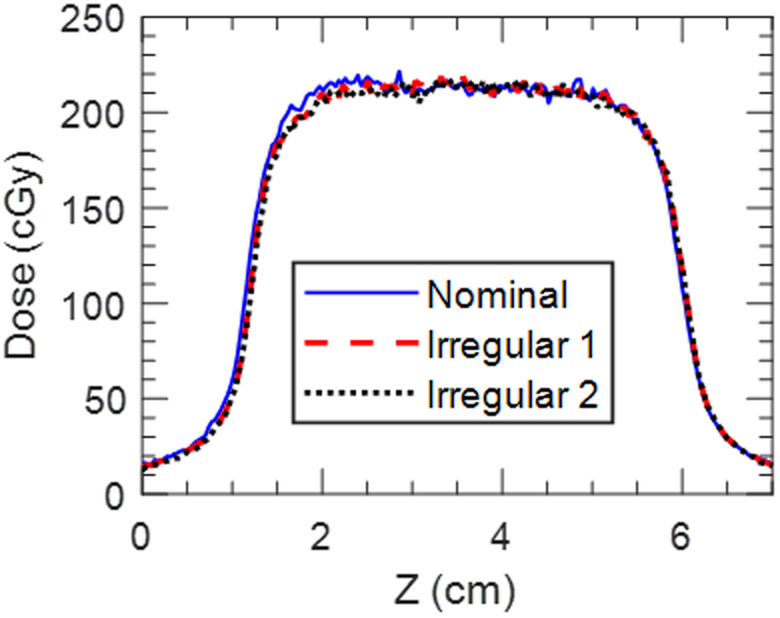
Superior–inferior profiles through the center of the target from film measurements of the 5 s/1 cm plan done with the nominal breathing trace and the two irregular traces shown in Fig. 9b.

Film measurements were acquired using the irregular traces shown in Fig. 9b to determine the robustness of the technique to non‐periodic breathing and changes in breathing between 4DCT acquisition and treatment. Fig. 14 shows superior–inferior profiles extracted from film measurements acquired with the nominal 5 s/1 cm and irregular breathing traces. The 2%/2 mm gamma pass rate when comparing the nominal and irregular trace measurements was 98%. This indicates that this technique is robust to variable breathing patterns.

## 4 DISCUSSION

The workflow presented here sequentially uses three different forms of pretreatment imaging to aid in setting and verifying the treatment amplitude gate thresholds. First, the use of 4DCBCT allows for a phase subset average CBCT reconstruction that corresponds to the planning phases. This means the initial 4DCBCT to planning CT match uses image sets that can be directly compared, allowing for automatching. Stepping through the 4DCBCT movie loop provides 3D visualization of motion. Next, the use of fluoroscopy enables the amplitude gate thresholds to be set based on breathing pattern and target motion at time of treatment. Since the gate thresholds are set based on when the target or surrogate is inside the tracking structure, changes in breathing pattern or target motion from time of simulation or previous fractions can be accounted for. The final gated CBCT provides confirmation of correct patient setup and respiratory gate thresholds. The flexibility of this technique allows it to be robust to variable breathing and target motion throughout the treatment process.

While the pretreatment imaging workflow was designed to sequentially use 4DCBCT, fluoroscopy, and gated CBCT, there are several variations depending on the case. For long course treatments, we often bypass 4DCBCT and fluoroscopy and start with the gated CBCT once respiratory motion and amplitude gate thresholds have been found to be reproducible over the first several fractions. A weekly 4DCBCT has been found to be useful in our clinic to explicitly verify target motion in such cases. For cases where the target is not visible on fluoroscopy and there is not a suitable surrogate, such as small or central lung tumors, daily motion is solely evaluated from the 4DCBCT. This motion evaluation is then used to estimate the amplitude gate thresholds corresponding to the phases in which the target is inside the tracking structure. When the target is not distinguishable on 4DCBCT imaging and there is no suitable surrogate, the amplitude gate is simply estimated considering the planning phases. This is most common in abdominal cases without fiducials that are located far from the diaphragm. For these cases, the iterative CBCT reconstruction algorithm,^23^ an additional add‐on TrueBeam imaging feature, has been useful to improve image quality and target visibility on the amplitude‐gated CBCT. For the latter two instances where the target is not visible on fluoroscopy and the amplitude gate thresholds are estimated from either 4DCBCT motion analysis or the planning phases, the phase dial in the TrueBeam software can be used to correlate the current respiratory trace with phase. As you step through the respiratory trace, the phase dial displays the phase at a given point of the trace, allowing the user to set amplitude gate thresholds that correspond to the desired phase range. The final verification of amplitude gate thresholds is always through the amplitude‐gated CBCT. Since the amplitude‐gated CBCT is acquired under the same conditions that treatment is delivered, if the match between amplitude‐gated CBCT and the planning CT is acceptable the gate thresholds are deemed to be appropriate. The gated CBCT is evaluated carefully to ensure that anatomy is consistent with the planning CT. If anatomy appears enlarged or blurred compared to the planning CT, this indicates that the amplitude gate is too wide and the gate thresholds are adjusted.

The use of 4DCBCT and fluoroscopy in the daily pretreatment imaging process leads to an increase in patient imaging dose compared to non‐gated treatments. However, the trade‐off between imaging dose and the decreased treatment volumes achievable with accurately delivered gated treatments must be considered. Published guidance in this area recommends practicing ALARA and making physicians aware of the associated increase in imaging dose.^24^ Varian reports that the weighted CT dose index (CTDI_w_) for a 4DCBCT scan acquired with default parameters is 10 mGy, compared to 4 mGy for a regular thorax 3DCBCT.^25^ For reference, 16 mGy is reported for a pelvis 3DCBCT with default parameters. Gated CBCT scans deliver more dose than the equivalent non‐gated scan since the frame rate is kept constant while the gantry is accelerating. This increase in dose is dependent on breathing period and duty cycle. For a typical breathing period of 5 s and duty cycle of 50%, Varian states that the CTDI of a gated scan increases by 9% compared to the non‐gated scan.^25^

By definition, gated treatments take longer than regular treatments to deliver since radiation is only delivered during a subset of the respiratory cycle. Due to the increased amount of imaging and the need to set amplitude gate thresholds, it is common for first fraction treatments to take approximately 30–40 minutes at our institution. Subsequent fractions once the gate has been set are often shorter and we have completed SBRT treatments with the entire imaging workflow in less than 20 minutes. Treatment time is largely dependent on the regularity of the patient’s breathing.

A challenge with the delivery of amplitude‐gated free breathing treatments is breathing irregularity during treatment, particularly negative drifts in baseline that result in inhale peaks being inside the respiratory gate. It has been shown that baseline drifts can indicate a change in internal target position.^26^ Therefore, our conservative policy in implementing this technique is to require reimaging when the breathing trace is relearned during treatment delivery to verify the relationship between the respiratory surrogate (TrueBeam reflector block) and internal anatomy.

The TrueBeam system automatically relearns the breathing trace after any couch movement greater than 2 mm, whether it be due to shifts applied from imaging or couch centering. If the patient's breathing pattern changes or is irregular during the relearning period, there is the potential to re‐baseline the respiratory trace at a different level compared to previous imaging. Therefore, our implementation of this technique requires that the last form of imaging prior to starting or resuming treatment is a gated CBCT where shifts are not required. Isocenter is placed to ensure that a gated CBCT can be acquired without centering the couch.

It has been demonstrated that patient coaching during treatment can improve breathing regularity and treatment reproducibility.^27^, ^28^ There are several audiovisual coaching options available within the TrueBeam system. Visual coaching can be provided in the form of a slider bar or movement along a path to indicate breathing amplitude. Audio instruction is also available to instruct patients when to inhale and exhale. Both audio and visual feedback settings can either be set manually or tied to the patient’s breathing pattern during the learning period. While these features have not been extensively explored at our institution, this could be used to help patients that do not naturally have a regular respiratory pattern.

While this work uses a TrueBeam linac, the developed workflow can be applied to other systems/vendors with similar capabilities, namely 4DCBCT, respiratory‐correlated fluoroscopy, and gated CBCT. A particularly important aspect to consider when using other systems is the convention by which the 4DCT and 4DCBCT phases are binned to ensure that the phase subset average CBCT appropriately corresponds to the planning phase subset average CT. The commissioning process described here could be undertaken with other motion phantoms. The phantom used in this work was limited to superior–inferior motion, but it could be advantageous to use a phantom capable of 3D target motion to better represent anatomical motion.

## 5 CONCLUSION

The free breathing respiratory‐gated treatment program described in this work uses multiple imaging modalities in a logical and sequential pretreatment imaging workflow. 4DCBCT and fluoroscopy imaging allow for daily evaluation of motion and determination of amplitude gate thresholds. The commissioning process established the accurate end to end delivery of these treatments for multiple respiratory patterns and across different users.

## AUTHOR CONTRIBUTIONS

Susannah Hickling, Andrew Veres and Michael Grams conceptualized the work and performed commissioning measurements. All authors contributed to data analysis and interpretation. Susannah Hickling prepared the manuscript. All authors critically revised the work and have given approval for the submission of this manuscript.

## CONFLICT OF INTEREST

No conflicts of interest.

## Data Availability

The data that support the findings of this study are available from the corresponding author upon reasonable request.
